# Implementing School-Based Mental Health Services: A Scoping Review of the Literature Summarizing the Factors That Affect Implementation

**DOI:** 10.3390/ijerph19063489

**Published:** 2022-03-15

**Authors:** Anne Richter, My Sjunnestrand, Maria Romare Strandh, Henna Hasson

**Affiliations:** 1 Procome Research Group, Department of Learning, Informatics, Management and Ethics, Medical Management Centre, Karolinska Institutet, 171 77 Stockholm, Sweden; my.sjunnestrand@ki.se (M.S.); maria.romare.strandh@kbh.uu.se (M.R.S.); henna.hasson@ki.se (H.H.); 2 Unit for Implementation and Evaluation, Center for Epidemiology and Community Medicine, Region Stockholm, 171 29 Stockholm, Sweden; 3 Reproductive Health Research Group, Department of Women’s and Children’s Health, Uppsala University Hospital, 751 85 Uppsala, Sweden

**Keywords:** implementation, school-based mental health services, mental health, scoping review

## Abstract

Background: Mental illness in children and youths has become an increasing problem. School-based mental health services (SBMHS) are an attempt to increase accessibility to mental health services. The effects of these services seem positive, with some mixed results. To date, little is known about the implementation process of SBMHS. Therefore, this scoping review synthesizes the literature on factors that affect the implementation of SBMHS. Methods: A scoping review based on four stages: (a) identifying relevant studies; (b) study selection; (c) charting the data; and (d) collating, summarizing, and reporting the results was performed. From the searches (4414 citations), 360 were include in the full-text screen and 38 in the review. Results: Implementation-related factors were found in all five domains of the Consolidated Framework for Implementation Research. However, certain subfactors were mentioned more often (e.g., the adaptability of the programs, communication, or engagement of key stakeholders). Conclusions: Even though SBMHS differed in their goals and way they were conducted, certain common implementation factors were highlighted more frequently. To minimize the challenges associated with these types of interventions, learning about the implementation of SBMHS and using this knowledge in practice when introducing SBMHS is essential to achieving the best possible effects with SMBHSs.

## 1. Background

Mental illness in children and youths has become a public health concern. Symptoms can range from mild and short-term problems, such as mild anxiety or depressive symptoms, to more severe and long-term forms of diagnosed anxiety disorders or major depression [1]. An estimated 12–30% of school-age children suffer from mental illness of sufficient intensity to adversely affect their education [2]. The vulnerability to mental illness is highest during childhood and adolescence [3]. Within the last decade, an increase in diagnoses related to mental ill-health has been noted [4]. An estimated 50% of all mental illnesses begin before the age of 14, and three-quarters of mental ill-health occurs before the age of 25 [5].

Tremendous social costs result from the consequences of leaving mental ill-health in children and youths untreated. These consequences may range from poor educational attainment, compromised physical health, substance abuse, juvenile delinquency, and unemployment to even premature mortality (e.g., suicide [6,7,8]). In line with that, cost-benefit analyses of mental health programs have found these programs result not only in economic productivity gains but also improved health [9,10].

Despite this, mental ill-health of children and youths is often not identified and treated in a timely way. Estimations show that up to 75% of students suffering from mental ill-health receive inadequate treatment or are not treated at all [11,12]. Consequently, mental ill-health often manifests in adulthood [5,13], which is unfortunate because many children and youths have originally mild or moderate symptoms [14]), and thus early identification and prevention can have beneficial effects [12,14,15]. Hence, there is an unmet need for mental health services for children and youth.

## 2. Mental Health Services Provided in Schools

Education and health are closely interlinked; school is important for one’s social and emotional development, and, therefore, school has an effect on health [16]. Moreover, due to compulsory school attendance, the majority of children and youths spend a considerable amount of time in schools, making schools an ideal environment to provide timely and convenient access to mental health services, including early identification, prevention, and interventions to prevent the escalation of mental ill-health [17]. In addition, providing services related to mental ill-health within the school setting has additional benefits such as cost efficiency and good accessibility to the services [18].

While school-based mental health services (SBMHS) may vary widely in focus, format, provider, and approach [19], they are all united in the fact that schools collaborate with health services to provide support for children and youths who are at risk of or have experienced mental ill-health. An SBMHS encompasses “any program, intervention, or strategy applied in a school setting that was specifically designed to influence students’ emotional, behavioral, and/or social functioning” [20](pp.224).

Even though services related to mental ill-health can be found outside the school setting, these community mental health services are often underutilized. For example, Kauffman, [21], Langer et al. [22], and Merikangas et al. [23] showed only 20% of children and youths received help to address their needs related to mental health, whereas Armbruster and Fallon [24], and McKay et al. [25] showed the help children and youths receive is often prematurely ended. Instead, SBMHS seem to resolve some of the known barriers that prevent access to mental health services for children and youths, such as lack of insurance, shortage of medical or psychological mental health professionals, mental health stigma, or the lack of transportation opportunities [26].

The effectiveness of SBMHS has been studied in several reviews and meta-analyses. In general, mental health programs through SBMHS were found to have a positive effect on emotional and behavior problems [20]. Hoagwood and Erwin [27] identified three types of services that had a clear impact (i.e., cognitive behavioral techniques, social skills training, and teacher consultation models). Other studies evaluating multifaceted and multilevel interventions showed improvements to mental health outcomes [28,29]. However, Caldwell et al. [30], focusing on SBMHS at secondary schools for youths with depression and anxiety, found limited evidence for their effectiveness. Fazel et al., [31] suggested these results might be premature and that long-term follow-ups should be applied to investigate effectiveness. Systematic reviews on the effect of SBMHS on specific target groups such as primary school children [32] or elementary school children [33] showed positive effects on their mental health. To conclude, even though these studies generally indicate positive effects of SBMHS, general conclusions are made difficult by the heterogeneity of interventions and evaluation designs used [34].

Besides the different definitions, the variety of programs included under the SBHMS umbrella, and the different designs for evaluation, these programs are complex, which also makes the implementation process potentially challenging. For example, Rones and Hoagwood [20] identified some features associated with the implementation that are important for the maintenance and sustainability of SBHMS programs (e.g., including various stakeholders, using different modalities, and integrating the intervention into the regular classroom curriculum). Without shedding more light on the implementation of SBMHS, there can be a risk of drawing false conclusions about the effectiveness of the programs. For instance, the lack of effects can be due to poor implementation instead of a failure of the theory underpinning the program [35]. In line with this reasoning, there has been a call to provide more clarity on the implementation of SBMHS [20,36].

## 3. Aim of the Review

This scoping review aimed at synthesizing the literature on the implementation of SBHMS. By doing so, we aim to increase the understanding of the systemic conditions and factors that affect the implementation of SBHMs.

The following research question will be addressed: Which factors are important for the implementation of school-based mental health services (SBMHS)? To systematize the findings, the factors relevant for implementing SBMHS will be structured according to Consolidated Framework for Implementation Research (CFIR) that differentiates between characteristics of the intervention and individuals using the intervention, the inner and outer context as well as the process of implementing.

## 4. Method

### 4.1. Study Design

To address the study aim, we performed a scoping review to identify barriers and enablers of the implementation of SBHMs. This method was chosen to provide a broad overview of implementation-related factors for SBHMs [37]. We followed the procedure outlined by Arksey and O’Malley [38]. After identifying the research question we (1) identified relevant studies, (2) selected studies, (3) charted the data, and (4) collected, summarized, and reported the results. Steps 1–3 are described in the Section 4, whereas Step 4 is presented in the Section 5.

### 4.2. Identify Relevant Studies

The search was conducted on 7 May 2019. The search strategy was developed in collaboration with a team of informatics experts from the university library at Karolinska Institutet. Based on several example papers focusing on SBHMs, and in discussion with representatives from the Swedish Public Health Agency and the Swedish Association of Local Authorities and Regions, potential keywords were identified. The search strategy included conducting searches in four databases: Medline, Eric, PsycINFO, and Web of Science (see Appendix A). Articles published up to May 2019 were included in the search. The informatics team provided a full list of references after duplicates had been removed. Articles were also found and added through manual searches based on recommendations.

Simultaneous with developing the search strategy, eligibility criteria for relevant studies were defined [38]. To be included, studies were required to focus on SBMHS and to have been conducted through a collaboration of school staff together with staff from social services and/or health-care services. The interventions had to address children and youths’ mental health and be published in English or a Scandinavian language. In addition to peer-reviewed journals, reports, and dissertations were included. Mental health was defined broadly and based on a definition by the Swedish Committee on Child Psychiatry [39], where mental ill-health is children’s lasting symptoms that prevent them from optimal functioning and development and that cause suffering. This included internalized mental health symptoms (e.g., anxiety, depressive symptoms, psychosomatic symptoms, eating disorder symptoms, and self-harming behaviors), externalized mental health symptoms (e.g., neuropsychiatric impairment, or behavioral problems), and indicators of psychological problems (e.g., school problems, trauma, or problems at home). Two reviewers tested the eligibility criteria on 40 articles from the final search. Inconsistencies in interpretations were discussed within the research group and with representatives from the Swedish Public Health Agency and the Swedish Association of Local Authorities and Regions, and thereafter modified to clarify the criteria.

### 4.3. Select Studies

All studies were screened to eliminate those that were not in line with the research question [38]. Rayyan, a software program that facilitates the screening process, was used [40]. Two authors (A.R. and M.R.S.) reviewed articles in addition to two research assistants. First, study titles and abstracts were evaluated based on the eligibility criteria in duplicate by two independent reviewers. Throughout the process, the reviewers met to discuss the eligibility criteria to confirm consensus, and modifications of the criteria were made to increase clarity (see Appendix B for eligibility criteria). The evaluation of titles and abstracts was finished in July 2019. The reviewers’ conflicting decisions were compared after completion. In the cases of inconsistencies in the decisions, the titles and abstracts were re-read and discussed in the reviewers’ group to reach a consensus. In addition, searches of the reference lists from relevant articles were also conducted to find potentially relevant articles (i.e., snowball search). These additional articles were screened in the same way as the original articles.

In the next step, the full texts of the included studies from the title and abstract evaluation were accessed for final inclusion. Three authors (A.R., M.R.S., and M.S.) and two research assistants reviewed articles in full text. As in the first step, the studies were assessed by two independent reviewers, and conflicting decisions were discussed to reach a consensus about the inclusion or exclusion of the study. The full-text evaluation was finished in November 2020.

### 4.4. Chart Data

In the next stage, key information from the included studies where charted [38]. The following information was collected from all included studies: (a) authors, (b) year of publication, (c) journal, (d) country of origin, (e) aim of the study, (f) study design, (g) method of data collection, (h) setting, (i) name of the intervention, (j) description of the intervention, (k) target groups for the intervention, (l) collaboration partners involved in conducting the intervention, (m) mental health challenge of the intervention target group, and (n) information about the implementation of the intervention. To test the chart template, all reviewers (A.R., M.R.S., M.S., and two research assistants) charted data from the same five included articles and compared the extracted data. Any inconsistencies were discussed, and the template was modified to increase clarity. Based on the information about the focus of the interventions and their target groups, these interventions were then categorized as universal, selective, or indicated. Universal interventions targeted all children, whereas selective interventions focused on risk groups and indicated interventions were provided to children and youths who were already struggling with their mental health.

To organize and categorize the information related to implementation, the Consolidated Framework for Implementation Research (CFIR, [41]) (for more information, see https://cfirguide.org, accessed on 10 February 2022) was used as a conceptual framework to structure the extracted information. CFIR clusters factors related to the implementation into five categories (intervention characteristics, inner setting, outer setting, characteristics of the individual, and implementation process).

The intervention characteristics describe the source of the intervention (i.e., perceptions of the source of the intervention), the evidence strength and quality that the intervention will have desired outcomes, the perceived advantage of implementing this intervention compared to other interventions (i.e., relative advantage), how complex and adaptable the intervention is as well as if the intervention can be tested small-scale first. The costs associated with the intervention as well as the perception about how the intervention is design, packaged, and presented also describe important intervention characteristics. The outer setting of the organization where the intervention is implemented is described by the prioritization of patient needs and the resources allocated to patient needs by the organization, in how fare the organization is part of a larger network (i.e., cosmopolitanism), if other organizations have implemented the intervention hence, there is peer pressure to also implement the intervention and if there are external policies and incentives that may affect the implementation of the intervention. The organization’s inner setting is described by structural characteristics (e.g., age, maturity or size of the organization), the nature and quality of networks as well as (in)formal communication within the organization as well as the culture (i.e., existing norms, values, or assumptions made by employees). Moreover, implementation climate (i.e., the capacity to implement change) is an important characteristic that is further differentiated in the tension for change (i.e., the perception that the current situation is intolerable and requires change), the compatibility of the intervention with existing workflows and norms, the relative priority the intervention is perceived to have, existing incentives and rewards that exist in the organization that affect the implementation process as well as the existence of a learning climate and clear goals and feedback related to the intervention. Another important factor of the inner context is the organization’s readiness for implementation (i.e., the commitment to the decision of implementing the intervention). Here the involvement and commitment of leaders (i.e., leadership engagement), the amount of dedicated resources for the intervention, as well as access to knowledge and information about the intervention and its implementation are important. Characteristics of individuals is another important factor according to CFIR. It is defined by individuals’ knowledge and beliefs about the intervention (e.g., attitudes towards the intervention), individuals’ belief in their own capacity to execute the intervention (i.e., self-efficacy), the phase of change individuals are in, but also individuals’ identification with the organization as well as personal characteristics such as motivation, value, or learning style (i.e., other personal attributes). The implementation process according to CFIR is categorized in a planning phase (e.g., schemes or methods that are developed in advance), engaging, executing, and reflecting and evaluating phase. Engaging, that is the involvement of appropriate individuals is further differentiated in the engagement of opinion leaders, (in)formally appointed implementation leaders, champions as well as external change agents.

Information from included studies that related to the implementation of SBMHS was extracted, and, in the next step, categorized based on the CFIR domains and their more specific subtopics.

## 5. Results

### 5.1. Included Studies

**Figure 1:**
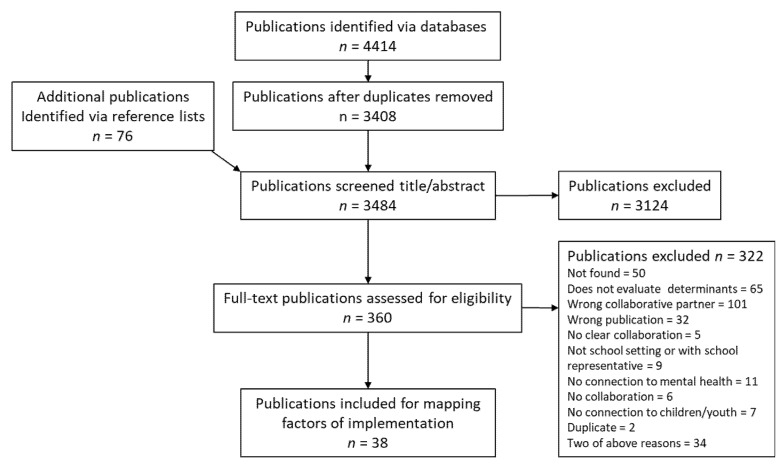
PRISMA flowchart for this review. Note: Eligibility criteria are presented in Appendix B.

The data search resulted in 4414 studies that were potentially relevant to the research question of this scoping review. After 1006 duplicates were removed, 3408 studies remained and were included in the screening of titles and abstracts, resulting in 360 potentially eligible studies. Of these, 38 studies were included in the review after full-text screening. The PRISMA flowchart (Figure 1) summarizes the screening steps and the numbers of included and excluded studies in each step of the screening.

### 5.2. Study Characteristics

The 38 studies were published between 1996 and 2018 (see Table 1). The majority of studies were conducted in the United States (*n* = 22), followed by Great Britain (*n* = 7), Australia (*n* = 5), and Canada (*n* = 2). One study was conducted in Finland and one in Sweden. The SBMHS included universal (*n* = 16), selective (*n* = 7), and indicated interventions (*n* = 14). For two SBMHS, the interventions seemed a mixture of selective and indicated interventions. Examples of universal interventions included providing mental health support services to all students or promoting school readiness by creating emotionally supportive classrooms. Examples of selective interventions were introducing a stress-reducing early intervention team to student cases with a risk of mental ill health or establishing collaborations between schools and mental health services to improve psychosocial functioning of students with learning disabilities at risk of mental ill health. Examples of indicated interventions were improving communications between caretakers of children with ADHD or implementing a social skills program to promote children’s cooperative skills and anger management. The majority of SBMHS (*n* = 12) focused on improving mental health in general, whereas others focused on more specific issues (e.g., ADHD *n* = 5, emotional and behavioral problems *n* = 4, or depression *n* = 3). Most studies described programs where schools collaborated with mental health services (*n* = 19), whereas seven programs included collaboration between schools and health-care services. Social services were involved in six programs.

### 5.3. Implementation Factors

A summary of factors related to the implementation of an SBMHS is presented in Table 2. More specific information about the factors influencing implementation in each of the included studies can be found in Table 3.

Generally, implementation-related information could be found for all five CFIR domains, but some of the subfactors in CFIR seemed to be particularly relevant to implementing SBMHS. Frequently named intervention characteristics were the adaptability of the intervention, the design quality and packaging of the intervention, and the costs associated with the intervention. For example, programs were often adapted to the content of the staff training, the way the treatment within the program was conducted, and the evaluation of the treatment compliance to fit to the local context [68]. Moreover, adaptation of the program to the local conditions and the target group was crucial [66]. One example of a concrete adaptation was to change the language used in the program so that students with diverse backgrounds could be reached [66]. Language and the way the program was packaged didactically was also identified in another study as culturally inappropriate and a hindrance to implementation of the program for certain minority groups [69]. Furthermore, the service range of the program as well as the facilities (e.g., rooms used for the programs) needed to be adapted based on the needs of the children and youths in that school [75]. Adaptability was more often mentioned when indicated programs were implemented compared to universal or selective programs.

Information related to the outer setting was mainly captured by the subfactors of cosmopolitanism and external policies and incentives. One reoccurring example related to external policies was the different compensation systems among cooperating actors [45,46]. Similarly, the different actors involved in the programs needed to gather consent from individual legal guardians of children and youths, as well as applying different principles of confidentiality, which also provided a challenge [60,65]. Having an established network with other organizations was also important for implementation. For example, when one needed to hire staff who could carry out the program, recruiting from organizations where established contacts existed facilitated the process (e.g., [52]).

Inner-setting factors that primarily were mentioned were the networks and communication, goals, and feedback, as well as the available resources that contributed to a readiness for the implementation. In particular, the need for an open dialogue between actors within the SBHMS was perceived as a cornerstone for developing trust and respect between actors [71]. A supportive administration department was highlighted as important for these multi-actor programs [80]. Dysfunctional communication could result in the loss of important information about students who participated in the program, which could affect the program and its outcomes negatively [55]. Clear goals and feedback as part of the implementation climate were also frequently mentioned. Particularly, different goals by various actors was highlighted as a potential challenge with SBMHS (e.g., [52]). Moreover, having sufficient resources such as suitable premises [51], the right technical aids [71], or adequate funding for the new initiative [81] was also important. In particular, studies on indicated interventions mentioned the availability of resources.

Regarding individuals’ characteristics is important for the implementation of SBHMs; in particular, actors’ knowledge of and belief in the program were mentioned. For example, when the actors involved strongly believed that the program would improve children’s mental health, staff’s motivation to work with the program increased [57].

When it comes to the process related to the program, engagement of key stakeholders and the participants in the interventions was frequently mentioned. For example, in Panayiotopoulos and Kerfoot [72], creating engagement with relevant actors was central to the implementation. These actors primarily included teachers and coordinators for nurses [69] and school management, as well as other staff at the school [59]. In another study, where a program for ADHD primarily focused on increasing the competence of physicians and teachers, the program did not achieve engagement of the targeted group, which affected the program’s effectiveness [79].

## 6. Discussion

Due to the increasing number of children and youths who are at risk of, and have experienced, mental ill-health, the efficient implementation of countermeasures such as SBMHS is essential. Therefore, this scoping review synthesized the available research on factors that influence the implementation of SBMHS. From 38 studies, information related to the implementation of SBMHS was gathered and structured. SBMHS have incorporated a variety of programs spanning from universal programs that target all students and aim at improving children and youths’ general mental well-being to programs that target specific individuals, either who were at risk for mental ill-health or who experienced mental ill-health. In addition, the SBMHS also varied in their focuses (i.e., the issues they primarily addressed). Whereas the universal programs focused on increasing general mental health or more specific facets of it (e.g., emotional or behavioral problems), the selective and particularly the indicated programs often addressed narrower topics (e.g., ADHD or depression). Most studies were conducted in English-speaking countries. Implementation-related factors of SBMHS for all five CFIR domains (i.e., intervention characteristics, outer setting, inner setting, characteristics of the individual, and process) were identified. However, information was primarily found around three of the five domains (i.e., intervention characteristics, inner setting, and process), and certain subfactors were mentioned more frequently than others were (i.e., design quality and packaging, adaptability, networks and communication, readiness for the implementation through available resources, engaging key stakeholders, and innovation participants).

### 6.1. Adapting of the Interventions

The design and packaging of the intervention was an often-mentioned factor and was often related to the adaptability of the intervention to the local context. Hence, for SBMHS implementation, being able to tailor a specific intervention to the needs and circumstances of the school and other actors involved was perceived important. Generally, adaptations have been discussed in relation to the fidelity of interventions, which presents the degree to which an intervention is carried out based on how it was described and originally tested when developed [82,83]. Fidelity has been an important factor in intervention implementation and is often studied as an implementation outcome [84]. However, real-world practice has shown that adaptations to interventions such as evidence-based interventions (EBIs) are in the majority of cases the rule rather than the expectation [85,86,87]. In recent years, adaptation has been discussed more frequently, but not as the opposite of fidelity as done before (e.g., [88]). Rather, adaptation is discussed in terms of how fidelity and adaptation can coexist when the core components of the intervention are preserved [89,90,91] and are necessary so that EBIs can result in value for all stakeholders when, for example, EBIs are implemented [92,93,94]. Examples of reasons for adaptation include increasing the fit between the intervention and context and being able to address multiple diagnoses or balance different outcomes [94]. Intervention strategies that aim at increasing this intervention–context fit could include community–academic partnerships, so that intervention developers and practitioners who shall work with the interventions collaboratively design the process [95]. Central is the transparency of adaptations, hence the conscious decisions and documentation about what is adapted, as well as how and for what reason, to avoid adaptation neglect, which may lead to the removal of the intervention’s central components, thereby threatening the intervention’s effectiveness [94]. In the case of SBMHS, where adaptations and the fit of existing design and packaging of programs seemed central, adaptations regarding the target groups or local conditions at schools were most relevant. However, another potential adaptation may concern implementing programs from other countries and hence other cultural settings [96].

### 6.2. Internal Collaboration and between Actors

SBMHS are essentially the collaboration of various actors who are relevant to children and youths’ mental health; that is, health-care providers, social-care providers, and schools. These three actors ultimately represent different organizations, which also means different primary goals, different ways of working, different cultures, and, potentially, different laws to which they relate. These organization-specific factors may represent challenges to smooth communication between actors when it comes to SBMHS, and they ultimately might make it harder to implement SBMHS successful. Organizational factors have been found to be critical for the successful implementation of evidence-based practices [97,98]. However, studies predominantly focus on one organization [99]; hence, the interorganizational alignment that may be of relevance for initiatives such as SBMHS has not received much research attention [99]. In line with the scarce empirical findings, theoretical frameworks also tend to focus on the one organizational setting. For example, in CFIR [41], organizational factors are categorized under the inner-setting domain. However, for SBMHS, the inner setting that may affect implementation is essentially several organizations’ inner settings. An exception is the Exploration, Preparation, Implementation, and Sustainment framework, which in addition to the interorganizational context, also includes only a few details. Our results indicate that communication, which might be an essential part of interorganizational collaboration, is important for SBMHS implementation. In the future, the interorganizational alignment of organizational constructs [99] should be studied more closely. Closely related to the communication aspect, resource availability for the implementation of SBMHS was often named. Resource availability could be a sign of the overall prioritization of the intervention. However, schools have limited financial resources, and often staff already experience high demands [100] This might indicate that schools that introduce SBMHS might need to conduct a thorough analysis beforehand to understand what is required for the intervention to be feasible in this context.

Stakeholder engagement is central to successful implementation in general [101,102], and, of course, relevant to specific programs that are provided within SBMHS, e.g., school-based intervention for trauma [103]. Potential stakeholders relevant for SBMHS are district and school administrators, mental-health service providers, and educators, as well as students and their families. A particular focus should be placed on gaining their buy-in [104] to make an implementation successful. Continuous stakeholder engagement could also increase communication and facilitate making decisions related to adaptations and their documentation and evaluation.

### 6.3. Implications for Implementing SBMHS

Taken together, this scoping review can be used as a resource and starting point for schools and their collaboration partners that aim at implementing SBMHS in the future. Relevant factors for implementation are highlighted here that can be incorporated and covered when planning the implementation process. One suggestion for successful implementation is the use of multifaceted implementation strategies [105]. Schools could use the findings of this scoping review as guidance when planning SBMHS implementation strategies, which may increase the chances that an SBMHS results in the intended effects (i.e., an improvement in children and youths’ mental health). In addition, this study may also contribute to scholars placing more emphasis on the implementation process (i.e., its planning, execution, and evaluation). Process evaluation might be particularly important to increase our understanding of which implementation factors are essential for certain interventions [106]. Ultimately, increased focus on implementation sheds more light on the dilemma of theory failure versus implementation failure when it comes to understanding results from SBMHS evaluations.

## 7. Limitations

The limitations of this scoping review should be acknowledged. This scoping review includes studies that were published until May 2019. Later studies were not included as the pandemic most likely affected the educational system differently in different countries due to the measures and contract restriction that were introduced. Hence, implementation factors that can be found in studies conducted during the pandemic might therefore primary be a representation of the pandemic measure each country has introduced and might therefore not be comparable to a non-pandemic situation. Future studies should investigate SBMHS and mental health of children and youth in future studies further. In addition, most studies included did not have an explicit focus on studying the implementation of SBMHS. Therefore, we might only have captured the most relevant factors that affected SBMHS implementation that therefore often were mentioned in the Section 6. We also chose to define SBMHS in this paper as the collaboration of at least two actors, with schools being one and health care or social services the other one. This might have led to the exclusion of programs that have other constellations of collaboration partners. Based on program aims (i.e., improving mental health), we chose schools, health care, and social services as the central actors to be considered. The majority of the included studies were conducted in English-speaking countries, predominantly the United States, and only two were conducted in Nordic countries. However, schools, health-care services, and social services have major organizational differences compared to their respective counterparts in different countries. Hence, generalizability of results might be limited. However, certain implementation-relevant factors have been named in a variety of studies, which indicates that those seem to be important beyond the national specificities of the school, health care, and social service system.

## 8. Conclusions

This scoping review demonstrated that specific implementation factors seem to be more important in the implementation of SBMHS. Besides the need to study the implementation process explicitly, valuable practical guidance can extracted from this scoping review when new SBMHS are planned or existing services optimized.

## Figures and Tables

**Table 1:** Information about the studies.

Authors, Year	Country	Data Collection	Target Intervention Group	Participating Actors	Type of Issue	Intervention Name/Goal	Intervention Type
Anderson-Butcher et al. [42]	USA	Quantitative	Students in 3rd, 6th, 8th, and 12th grades	School, health-care providers, social service	Students at risk for poor academic and developmental outcomes	Ohio Community Collaboration Model for School Improvement (OCCMSI). Help schools and districts expand improvement efforts for at-risk children.	Selective
Atkins et al. [43]	USA	Quantitative	School teachers in urban, deprived areas	School and mental health services	ADHD	Increase the use of practices for children with ADHD.	Selective
Axberg et al. [44]	Sweden	Quantitative	Youth with externalizing problems	School, mental health services	Externalizing behavior	Marte Meo (MM) and Coordination Meeting (CM). Help children with externalizing problems and help their families.	Indicated
Baxendale et al. [45]	USA	Qualitative	Youth with communication needs	School, health care	Communication disorder	The Social Communication Intervention Project (SCIP). Enhance communication skills.	Indicated
Bellinger et al. [46]	USA	Quantitative	Children (ages 3–8) who experienced frequent noncompliance at home and school	School, mental health services	Behavioral and emotional problems	Conjoint Behavioral Consulting (CBC). Address student needs via evidence-based interventions, involve and engage families in their child’s education, and facilitate partnerships and build relationships between schools and families.	Indicated
Bhatara et al. [47]	USA	Qualitative	Teachers	School, mental health services, social services	ADHD	Swanson, Kotkin, Agler, M-Flynn and Pelham Scale-Teacher Version (T-SKAMP). Promote grading efficacy for children with ADHD.	Universal
Bruns et al. [48]	USA	Quantitative	All students at a public elementary school	School, mental health services	Emotional and behavioral problems	Expanded School Mental Health (ESMH). Provide school-based mental health services.	Universal
Capp [49]	USA	Qualitative	School students and staff and parents	School, mental health services	Diagnosable mental health disorders	Our Community, Our Schools (OCOS). Provide easy access to mental health promotion and treatment for students and their families, including access for those without insurance.	Universal
Clarke et al. [50]	UK	Mixed	School nurses and elementary school students, aged 10–11, in deprived areas	School, mental health services, and social services	General mental health issues	Facilitate accessible mental health support for young people, provide a problem- solving model for adolescents who have mental health issues, and support the role of school nurses by enhancing of their skills in mental health.	Universal
Fazel et al. [51]	UK	Quantitative	Refugee children and school staff	Schools and mental health services	Risk of emotional and behavioral problems	Provide a mental health service for refugees.	Selective
Fiester and Nathanson [52]	USA	Qualitative	School students	Schools and health-care providers	General mental health issues	Provide violence prevention and mental health services.	Universal
Foy and Earls [53]	USA	Qualitative	Community stakeholders, teachers, and parents	Schools and health-care providers	ADHD	Increase practice efficiency and improve practice standards for children with ADHD.	Indicated
Goodwin et al. [54]	USA	Quantitative	Children older than 5 years in child-care centers, preschools, or in a child-care provider’s home care	Schools, mental health services, and health-care providers	Emotional or behavioral problems	The Childreach program. Decrease violent and aggressive behavior in preschool-age children.	Selective
Hunter et al. [55]	UK	Qualitative	Students in secondary education	Schools and mental health services	General mental health issues	Enhance the effectiveness of the interface between primary care and specialist CAMHS services.	Universal
Jaatinen et al. [56]	Finland	No info	Children and adolescence	Schools, mental health services, health-care providers, and social services	Mental health and psychosocial problems	Provide psychosocial support for schoolchildren via networking family counselling services.	Universal
Jennings et al. [57]	USA	Mixed	Youth in an urban school district and their families	Schools and mental-health services	General mental health issues	Dallas (Texas) public school initiative. Provide physical health, mental health, and other support services for students and their families.	Universal
Juszczak et al. [58]	USA	Quantitative	All children who visited a clinic or school mental-health service	Schools and health-care providers	General mental health issues	School-Based Health Centers. Facilitate access to care.	Universal
Khan et al. [59]	Australia	Qualitative	Secondary- school students	Schools, mental health services, and health-care providers	General mental health	MindMatters. Improve health, well-being, and education outcomes in secondary schools in south-west Sydney.	Selective
Kutcher and Wei [60]	Canada	Mixed	School students	Schools, mental-health services, health-care providers, and social services	General mental health services	The School-Based Pathway to Care Model. Enhance the collaboration between schools, health-care providers, and community stakeholders to meet the need for mental-health support for adolescents.	Universal
Li-Grining et al. [61]	USA	Quantitative	All caregiving adults (e.g., teachers) and children from a preschool	Schools, mental-health services, and social services	General emotional and behavioral issues	Chicago School Readiness Project (CSRP). Promote low-income young children’s school readiness by creating emotionally supportive classrooms and by fostering preschoolers’ self-regulatory competence.	Universal
Maddern et al. [62]	UK	Mixed	Children with severe emotional and behavioral problems and their parents	Schools and mental-health services	Severe emotional and behavioral problems	Promote children’s cooperative skills and anger management.	Indicated
Mcallister et al. [63]	Australia	Quantitative	13-year-old children in rural areas	Schools and mental-health services	Psychological distress	Icare-R. Promote mental health.	Universal
Mckenzie et al. [64]	UK	Quantitative	Students in a rural area and guidance staff	Schools and mental-health services	General mental health issues	Provide community-based school counselling services.	Universal
Mellin and Weist [65]	USA	Qualitative	Elementary/middle (combined in this district) and high school students	Schools and mental-health services	General mental health	Enhance collaboration between schools and mental health services.	Universal
Mishna and Muskat [66]	Canada	Mixed	Students with various social, emotional, and behavioral problems; their families; school peers; school personnel; and social workers	Schools, mental-health services, and social services	Learning disabilities and psychosocial problems	Improve the psychosocial functioning of high-risk students with learning disabilities and psychosocial problems and increase the understanding of their learning disability.	Selective
Moilanen and Med [67]	USA	Mixed	Students in grades 8 through 12, school personnel, and parents	Schools and mental-health services	Depression and suicide	Prevent depression and suicide within high schools and local communities	Universal/Indicated
Mufson et al. [68]	USA	Quantitative	Depressed youth	Schools, mental-health services, health-care providers, and social services	Depression	IPT-A. Reduce depressive symptoms and improve interpersonal functions.	Indicated
Munns et al. [69]	Australia	Qualitative	Primary school-aged children who experienced loss (such as a death in the family, parental divorce, or other painful transitions)	Schools and health-care providers	Traumatic events	The Rainbow program. Support children who have experienced traumatic events	Indicated
O’Callaghan and Cunningham [70]	UK	Mixed	Primary-age children, 8- to 11-year-old pupils	Schools and mental-health services	Anxiety, depression, or low self-esteem	Cool Connections. Decrease depression and the risk of suicide and improve self-perception.	Indicated
Owens et al. [71]	USA	Mixed	Students in kindergarten through 6th grade	Schools and mental-health services	ADHD	Youth Experiencing Success in School (YESS). Enhance the use of EBTs in schools, improve the academic and behavioral functioning of children, enhance home–school collaboration and support services for parents, and provide ongoing collaborative consultation for teachers.	Indicated
Panayiotopoulos and Kerfoot [72]	UK	Mixed	Pupils, their family, and school staff	Schools, mental-health services, and social services	School exclusion	A home and school support project (HASSP). Prevent school exclusions.	Indicated
Powell et al. [73]	USA	Quantitative	Students in grades 7 to 12	Schools and mental health services	Emotional and behavioral disorders and educational disabilities	Help students return to public-school settings as quickly as possible.	Indicated
Rosenblatt et al. [74]	USA	Quantitative	Special education students/students with SED	Schools and mental-health services	Severe emotional disturbance (SED)	Provide collaborative mental health and education services.	Indicated
Stanzel [75]	Australia	Qualitative	High school students in rural areas	Schools and health-care providers	General mental health	Outreach youth clinic (OYC). Promote better health for young people by ensuring coordination between schools and community health and support s ervices.	Universal
Vander Stoep et al. [76]	USA	Quantitative	6th-grade students, the majority in special-needs groups	Schools and mental-health services	Emotional distress	Developmental Pathways Screening Program (DPSP). Identify youth experiencing significant emotional distress who need support services.	Universal
White et al. [77]	USA	Quantitative	Students returning to school after a psychiatric hospitalization or other prolonged absence due to mental-health reasons and their families	Schools and mental-health services	General mental-health issues	Bridge for Resilient Youth in Transition. Support academic and clinical outcomes for high school students returning to school after a mental-health crisis.	Selective and indicated
Winther et al. [78]	Australia	Quantitative	All children from preparatory to grade 3 (ages 4–10 years), teachers, and parents	School, health care and mental-health services	Oppositional defiance disorder/conduct disorder (ODD/CD)	Royal Children’s Hospital, Child and Adolescent Mental Health Service and Schools’ Early Action Program. Address emerging ODD/CD.	Indicated
Wolraich et al. [79]	USA	Mixed	ADHD children and their caregivers, medical services, and teachers	Schools and health-care providers	ADHD	Improve communication between individuals who care for children with ADHD.	Indicated

Notes: Universal interventions targeted all children, whereas selective interventions focused on risk groups and indicated interventions were provided to children and youths who were already struggling with their mental health.

**Table 2:** Implementation factors related to SBMHS.

CFIR Domains	All Studies *n* = 38	Universal Interventions *n* = 17	Selective Interventions *n* = 7	Indicated Interventions *n* = 14
**I.** **INTERVENTION CHARACTERISTICS**	**47**	**17**	**8**	**22**
Intervention Source	-	-	-	-
Evidence Strength and Quality	3	-	2	1
Relative Advantage	2	1	-	1
Adaptability	11	2	2	7
Trialability	3	1	-	2
Complexity	2	2	-	-
Design Quality and Packaging	19	9	2	8
Cost	7	2	2	3
**II.** **OUTER SETTING**	**19**	**9**	**2**	**8**
Patient Needs and Resources	1	-	-	1
Cosmopolitanism	6	3	1	2
Peer Pressure	2	-	1	1
External Policy and Incentives	10	6	-	4
**III.** **INNER SETTING**	**62**	**30**	**12**	**20**
Structural Characteristics	4	1	2	1
Networks and Communications	17	9	3	5
Culture	6	4	1	1
Implementation Climate	-	-	-	-
- Tension for Change	-	-	-	-
- Compatibility	2	1	-	1
- Relative Priority	4	2	1	1
- Organizational Incentives	-	-	-	-
- Goals and Feedback	9	4	2	3
- Learning Climate	-	-	-	-
Readiness for Implementation	-	-	-	-
- Leadership Engagement	2	2	-	-
- Available Resources	16	5	3	8
- Access to Information	2	2	-	-
**IV.** **INDIVIDUALS’ CHARACTERISTICS**	**11**	**2**	**3**	**5**
Knowledge and Beliefs About the Innovation	9	2	2	4
Self-Efficacy	-	-	-	-
Individual Stage of Change	-	-	-	-
Individual Identification with Organization	-	-	-	-
Other Personal Attributes	2	-	1	1
**V.** **PROCESS**	**40**	**20**	**9**	**11**
Planning	5	5	-	-
Engaging	-	-	-	-
- Opinion Leaders	3	-	2	1
- Formally Appointed Internal Implementation Leaders	2	1	1	-
- Champions	-	-	-	-
- External Change Agents	1	-	1	-
- Key Stakeholders	17	10	3	4
- Innovation Participants	9	3	2	4
Executing	1	-	-	1
Reflecting and Evaluating	2	1	-	1

**Table 3:** Implementation-related information per study.

Reference	Process	Inner Setting	Outer Setting	Intervention Characteristics	Individuals’ Characteristics
Anderson-Butcher et al. [42]		Implementation Climate—Relative Priority Implementation Climate—Goals and Feedback		Adaptability	
Atkins et al. [43]	Engaging Opinion Leaders				
Axberg et al. [44]		Networks and Communications		Trialability Design Quality and Packaging Adaptability	
Baxendale et al. [45]	Reflecting and Evaluating Planning Engaging Innovation Participants	Implementation Climate— Compatibility Readiness for Implementation—Available Resources	External Policy and Incentives	Design Quality and Packaging Adaptability Evidence Strength and Quality	Knowledge and Beliefs
Bellinger et al. [46]		Readiness for Implementation—Available Resources	External Policy and Incentives	Cost Design Quality and Packaging	
Bhatara et al. [47]	Engaging Key Stakeholders		Cosmopolitanism	Design Quality and Packaging	
Bruns et al. [48]				Design Quality and Packaging	
Capp [49]	Engaging Key Stakeholders Engaging Innovation Participants	Readiness for Implementation—Available Resources		Design Quality and Packaging Cost	
Clarke et al. [50]	Engaging Key Stakeholders				
Fazel et al. [51]	Engaging Innovation Participants	Readiness for Implementation—Available Resources Networks and Communications	Peer Pressure	Evidence Strength and Quality	
Fiester and Nathanson [52]	Planning Engaging Key Stakeholders	Implementation Climate—Relative Priority Readiness for Implementation—Leadership Engagement Implementation Climate—Goals and Feedback Culture Readiness for Implementation—Available Resources	External Policy and Incentives Cosmopolitanism	Complexity	
Foy and Earls [53]	Engaging Key Stakeholders		External Policy and Incentives Cosmopolitanism		
Goodwin et al. [54]			Cosmopolitanism	Cost	Other Personal Attributes
Hunter et al. [55]	Engaging Key Stakeholders	Implementation Climate— Compatibility Readiness for Implementation—Access to Information Readiness for Implementation—Available Resources Implementation Climate—Goals and Feedback Culture Networks and Communications	External Policy and Incentives	Relative Advantage Trialability	
Jaatinen et al. [56]	Engaging Key Stakeholders	Networks and Communications			
Jennings et al. [57]	Engaging Innovation Participants Engaging Key Stakeholders	Networks and Communications	External Policy and Incentives		Knowledge and Beliefs
Juszczak et al. [58]			External Policy and Incentives		
Khan et al. [59]	Engaging Key Stakeholders Engaging Innovation Participants Engaging External Change Agent Engaging Formally Appointed Internal Implementation Leaders	Structural Characteristics Networks and Communications Culture Readiness for Implementation—Available Resources		Design Quality and Packaging Cost	Knowledge and Beliefs
Kutcher and Wei [60]	Reflecting and Evaluating Engaging Key Stakeholders	Networks and Communications Implementation Climate—Goals and Feedback	External Policy and Incentives	Adaptability Design Quality and Packaging	Knowledge and Beliefs
Li-Grining et al. [61]	Planning	Networks and Communications Culture		Complexity Design Quality and Packaging	
Maddern et al. [62]	Engaging Innovation Participants Engaging Key Stakeholders	Implementation Climate Readiness for Implementation—Available Resources Implementation Climate—Goals and Feedback Networks and Communications Structural Characteristics	Patient Needs and Resources Peer Pressure	Adaptability Design Quality and Packaging	
Mcallister et al. [63]		Implementation Climate—Relative Priority Networks and Communications		Design Quality and Packaging	
Mckenzie et al. [64]	Engaging Innovation Participants	Readiness for Implementation—Leadership Engagement Networks and Communications		Design Quality and Packaging	
Mellin and Weist [65]	Planning Engaging Key Stakeholders	Networks and Communications Structural Characteristics Readiness for Implementation—Available Resources Culture Implementation Climate—Goals and Feedback	External Policy and Incentives		Knowledge and Beliefs
Mishna and Muskat [66]	Engaging Opinion Leaders Engaging Key Stakeholders	Implementation Climate—Goals and Feedback Networks and Communications Structural Characteristics		Design Quality and Packaging Evidence Strength and Quality Adaptability	Knowledge and Beliefs
Moilanen and Med [67]	Engaging Key Stakeholders			Design Quality and Packaging	
Mufson et al. [68]	Engaging Innovation Participants	Readiness for Implementation—Available Resources		Adaptability Design Quality and Packaging	
Munns et al. [69]	Engaging Key Stakeholders	Readiness for Implementation—Available Resources Networks and Communications	Cosmopolitanism	Design Quality and Packaging Cost Adaptability	
O’Callaghan and Cunningham [70]		Networks and Communications		Design Quality and Packaging	
Owens et al. [71]	Planning Engaging Opinion Leaders Executing	Networks and Communications Implementation Climate—Goals and Feedback Readiness for Implementation—Available Resources	External Policy and Incentives	Trialability	Other Personal Attributes Knowledge and Beliefs
Panayiotopoulos and Kerfoot [72]	Engaging Key Stakeholders	Implementation Climate—Goals and Feedback		Adaptability	Knowledge and Beliefs
Powell et al. [73]				Adaptability	
Rosenblatt et al. [74]		Readiness for Implementation—Available Resources Culture			Knowledge and Beliefs
Stanzel [75]	Engaging Formally Appointed Internal Implementation Leaders	Networks and Communications Readiness for Implementation—Access to Knowledge and Information		Design Quality and Packaging Adaptability	
Vander Stoep et al. [76]		Readiness for Implementation—Available Resources	Cosmopolitanism	Cost	
White et al. [77]	Engaging Key Stakeholders	Readiness for Implementation—Available Resources Implementation Climate—Relative Priority			
Winther et al. [78]		Readiness for Implementation—Available Resources		Cost Design Quality and Packaging	
Wolraich et al. [79]	Engaging Innovation Participants			Relative Advantage	

## Data Availability

Not applicable.

## Appendix A. Search Strategy

Documentation of search strategies

University Library search consultation group

Databases:

Medline (Ovid)

Web of Science Core Collection (Clarivate)

PsycInfo (Ovid)

ERIC (ProQuest)

Total number of hits:

Before deduplication: 12,000

After deduplication: 8000

Comments:

### Appendix A.1. Medline

Interface: Ovid MEDLINE(R) and Epub Ahead of Print, In-Process and Other Non-Indexed Citations and Daily Date of Search: 5 May 2019 Number of hits: 1095 Comment: In Ovid, two or more words are automatically searched as phrases; i.e., no quotation marks are needed	Field labels exp/= exploded MeSH term /= non exploded MeSH term .ti,ab,kf. = title, abstract and author keywords adjx = within x words, regardless of order * = truncation of word for alternate endings
1. Mental Disorders/ 2. Mental Health/ 3. Psychopathology/ 4. Adjustment Disorders/ 5. Affective Symptoms/ 6. exp Mood Disorders/ 7. Depression/ 8. exp Anxiety Disorders/ 9. Anxiety/ 10. Fear/ 11. Panic/ 12. Performance Anxiety/ 13. exp “Feeding and Eating Disorders”/ 14. Compulsive Personality Disorder/ 15. Obsessive Behavior/ 16. Compulsive Behavior/ 17. Impulsive Behavior/ 18. Child Reactive Disorders/ 19. exp Aggression/ 20. exp Self-Injurious Behavior/ 21. Psychophysiologic Disorders/ 22. exp Somatoform Disorders/ 23. exp Sleep Wake Disorders/ 24. Abdominal Pain/ 25. exp Headache Disorders, Primary/ 26. Headache/ 27. Chronic Pain/ 28. Musculoskeletal Pain/ 29. Back Pain/ 30. Low Back Pain/ 31. exp “Attention Deficit and Disruptive Behavior Disorders”/ 32. Hyperkinesis/ 33. Child Behavior Disorders/ 34. Social Problems/ 35. Juvenile Delinquency/ 36. Social Behavior Disorders/ 37. Antisocial Personality Disorder/ 38. exp Substance-Related Disorders/ 39. Stress, Psychological/ 40. Quality of life/ 41. Personal satisfaction/ 42. Happiness/ 43. Pessimism/ 44. Self Concept/ 45. Body Image/ 46. Self Efficacy/ 47. Sense of Coherence/ 48. Adaptation, Psychological/ 49. Resilience, Psychological/ 50. ((mental or emotional) adj3 (disease* or disorder* or distress or health or illness* or ill health or illhealth or instabilit* or problem* or symptom*)).ti,ab,kf. 51. ((psychiatric or psychologic*) adj3 (disorder* or distress or ill health or illhealth or illness* or problem* or symptom*)).ti,ab,kf. 52. ((behavior or behaviour) adj2 (disorder* or problem* or symptom*)).ti,ab,kf. 53. (affective adj2 (disorde* or distress or problem* or symptom* or syndrome*)).ti,ab,kf. 54. (abdominal pain or abnormal psychology or adhd or adjustment disorder* or acrophobia or aggression or aggressive* or agoraphob* or anankastic or anorectic or anorexia* or antisocial or anxiety or anxious or attention defici* or attention problem* or avoidance or avoidant disorder* or back ache or back pain or binge eating or bulimi* or chronic pain or claustrophobia or compulsion or compulsiveness or compulsive or concentration problem* or delinquenc* or delinquent or depress* or despair or despondency or dysthymi* or eating problem* or externalising or externalizing or fear or frustrat* or headache or health complaint* or hopeless* or hyperactiv* or hyperkines* or impulsiv* or inattention or insomnia or internal distress or internalising or internalizing or intrusive thinking or intrusive thoughts or loneliness or lonely or mood or moods or musculoskeletal pain or musculoskeletal symptom* or neophobia or nervousness or norm breaking or obsess* or ophidiophobia or overanxious or pain disorder* or panic attack* or panic disorder* or perpetrator* or persistent pain or pessimism or pessimistic or phobia or phobic or psychopathology or psychosocial or psychosomatic* or recurrent pain or rule breaking or sadness or sad or school phobia or self-cut* or self-destructive* or self-harm* or self-injur* or suicide or sleep disturbance or sleep problem* or stomach ache or stomachache or social problem* or social withdrawal or unhapp* or worries or worry).ti,ab,kf. 55. ((alcohol or appetite or body dysmorphic or body image or cyclothymic or child reactive or dysthymic or eating or factitious or feeding or impulse control or sleep or somatoform or substance) adj1 disorder*).ti,ab,kf. 56. ((alcohol* or drug* or substance* or tobacco) adj3 (abuse or addiction or dependence or habituation or misuse or “use”)).ti,ab,kf. 57. (well-being or wellbeing or wellness or optimis* or cheerful* or contentment or elated or elation or joy or enjoyment or good feeling* or good mood or happiness or happy or satisfaction or quality of life or HRQoL or QoL or sense of coherence or resilience or coping).ti,ab,kf. 58. (positive adj3 (affect* or emotion* or mood*)).ti,ab,kf. 59. (self adj3 (concept* or perception* or acceptance or confidence or esteem* or image or efficacy or reliance or worth or compassion)).ti,ab,kf. 60. ((emotional* or event* or level* or life or perceiv*) adj3 stress*).ti,ab,kf. 61. or/1–60 62. Schools/ 63. (classroom* or highschool* or pupil* or school* or teacher* or grade-1 or grade one first-grade or 1st-grade or grade-2 or grade two or second-grade or 2nd-grade or grade 3 or grade-three or third-grade or 3rd-grade grade-4 or grade-four or fourth-grade or 4th-grade or grade-5 or grade five or 5th grade or fifth grade or grade-6 or grade-six or 6th grade or sixth grade or grade-7 or grade-seven or 7th grade or seventh grade or grade-8 or grade-eight or 8th grade or eight grade or grade-9 or grade-nine or 9th grade or ninth grade or grade-10 or grade-ten or 10th grade or tenth grade or grade-11 or grade-eleven or 11th grade or eleventh grade or grade-12 or grade-twelve 12th grade or twelfth grade).ti,ab,kf. 64. or/62–63 65. Adolescent/ 66. Child/ 67. (adolescen* or boys or child* or girls or juvenil* or minor* or offspring* or puberty or school-age* or teen* or young* or youth* or underage* or under age*).ti,ab,kf. 68. or/65–67 69. 64 and 68 70. School Health Services/ 71. School Nursing/ 72. ((classroom* or highschool* or pupil* or school* or teacher*) adj3 (based or environment* or intervent* or implement* or setting*)).ti,ab,kf. 73. (school* adj2 (elementary or high or middle or primary or secondary)).ti,ab,kf. 74. or/69–73 75. Patient Care Team/ 76. Intersectoral Collaboration/ 77. Cooperative Behavior/ 78. Interinstitutional Relations/ 79. Interprofessional Relations/ 80. Interdisciplinary Communication/ 81. ((cross-agenc* or crossagenc* or cross-disciplinar* or crossdisciplinar* or cross-institutional* or crossinstitutional* or cross-organi#ational* or crossorgani#ational* or cross-professional* or crossprofessional* or crosssectoral* or cross-sectoral* or inter-agenc* or interagenc* or inter-disciplinar* or interdisciplinar* or inter-institutional* or interinstitutional* or inter-organi#ational* or interorgani#ational* or inter-professional* or interprofessional* or inter-sectoral* or intersectoral* or multi-agenc* or multiagenc* or multi-disciplinar* or multidisciplinar* or multi-institutional* or multiinstitutional* or multi-organi#ational* or multiorgani#ational* or multi-professional* or multiprofessional* or multi-sector* or multisector* or trans-agenc* or transagenc* or trans-disciplinar* or transdisciplinar* or trans-institutional* or transinstitutional* or trans-organi#ational* or transorgani#ational* or trans-professional* or transprofessional* or trans-sectoral* or transsectoral*) adj6 (care or collaborat* or communicat* or cooperat* or health care or intervention* or mental health or partnership* or program* or relation* or team* or strateg*)).ti,ab,kf. 82. (professional* adj3 (collaborat* or coordinat* or cooperat* or partnership* or teamwork)).ti,ab,kf. 83. ((collaborat* or coordinat* or cooperat*) adj6 (behavior* or behaviour* or health or intervention* or mental health or program* or school* staff* or school* nurs* or strateg*)).ti,ab,kf. 84. or/75–83 85. Child Welfare/ 86. Child Psychiatry/ 87. Adolescent Psychiatry/ 88. Health Services/ 89. Adolescent Health Services/ 90. Community Health Services/ 91. Community Health Nursing/ 92. Community Mental Health Services/ 93. Emergency Services, Psychiatric/ 94. exp Mental Health Services/ 95. Psychiatric Nursing/ 96. exp Community Psychiatry/ 97. Psychiatric Rehabilitation/ 98. exp Social Work/ 99. Primary Health Care/ 100. ((emergenc* or health or psychiat* or psycholog*) adj3 (care or nursing or practice* or service*)).ti,ab,kf. 101. (adolescent psychiatry or assertive community treatment or child guidance or child psychiatry or child welfare or community psychiatry or healthcare or mental health center* or mental health clinic* or mental health rehabilitation or primary care or psychiatric rehabilitation or psycholog* rehabilitation or psychosocial rehabilitation or social psychiatry or social service* or social work* or support* or treat*).ti,ab,kf. 102. or/85–101 103. Program Evaluation/ 104. Health Plan Implementation/ 105. (barrier* or determinant* or effect* or evaluat* or facilitat* or factor* or implement* or intervent* or predict* or program*).ti,ab,kf. 106. or/103–105 107. 61 and 74 and 84 and 102 and 106 108. limit 107 to (danish or english or norwegian or swedish) 109. remove duplicates from 108	

### Appendix A.2. Web of Science Core Collection

Interface: Clarivate Analytics Date of Search: 3 May 2019 Number of hits: 850	Field labels TS/TOPIC = title, abstract, author keywords and Keywords Plus NEAR/x = within x words, regardless of order * = truncation of word for alternate endings
#1. TS=((“mental” or “emotional”) NEAR/2 (disease* or disorder* or “distress” or “health” or illness* or “ill health” or “illhealth” or instabilit* or problem* or symptom*)) #2. TS=((“psychiatric” or psychologic*) NEAR/2 (disorder* or “distress” or “ill health” or “illhealth” or illness* or problem* or symptom*)) #3. TS=((“behavior” or “behavior”) NEAR/1 (disorder* or problem* or symptom*)) #4. TS=((“affective” NEAR/1 (disorde* or “distress” or problem* or symptom* or syndrome*))) #5. TOPIC: ((“abdominal pain” or “abnormal psychology” or “adhd” or “adjustment disorder*” or “acrophobia” or “aggression” or aggressive* or agoraphob* or “anankastic” or “anorectic” or anorexia* or “antisocial” or “anxiety” or “anxious” or “attention defici*” or “attention problem*” or “avoidance” or “avoidant disorder*” or “back ache” or “back pain” or “binge eating” or bulimi* or “chronic pain” or “claustrophobia” or “compulsion” or “compulsiveness” or “compulsive” or “concentration problem*” or delinquenc* or “delinquent” or depress* or “despair” or “despondency” or dysthymi* or “eating problem*” or “externalizing” or “externalizing” or “fear” or frustrat* or “headache” or “health complaint*” or hopeless* or hyperactiv* or hyperkines* or impulsiv* or “inattention” or “insomnia” or “internal distress” or “internalizing” or “internalizing” or “intrusive thinking” or “intrusive thoughts” or “loneliness” or “lonely” or “mood” or “moods” or “musculoskeletal pain” or “musculoskeletal symptom*” or “neophobia” or “nervousness” or “norm breaking” or obsess* or “ophidiophobia” or “overanxious” or “pain disorder*” or “panic attack*” or “panic disorder*” or perpetrator* or “persistent pain” or “pessimism” or “pessimistic” or “phobia” or “phobic” or “psychopathology” or “psychosocial” or psychosomatic* or “recurrent pain” or “rule breaking” or “sadness” or “sad” or “school phobia” or self-cut* or self-destructive* or self-harm* or self-injur* or “suicide” or “sleep disturbance” or “sleep problem*” or “stomach ache” or “stomachache” or “social problem*” or “social withdrawal” or unhapp* or “worries” or “worry”)) #6. TS=(((“alcohol” or “appetite” or “body dysmorphic” or “body image” or “cyclothymic” or “child reactive” or “dysthymic” or “eating” or “factitious” or “feeding” or “impulse control” or “sleep” or “somatoform” or “substance”) NEAR disorder*)) #7. TS=(((alcohol* or drug* or substance* or “tobacco”) NEAR/2 (“abuse” or “addiction” or “dependence” or “habituation” or “misuse” or “use”))) #8. TOPIC: ((well-being or “wellbeing” or “wellness” or optimis* or cheerful* or “contentment” or “elated” or “elation” or “joy” or “enjoyment” or “good feeling*” or “good mood” or “happiness” or “happy” or “satisfaction” or “quality of life” or “HRQoL” or “QoL” or “sense of coherence” or “resilience” or “coping”)) #9. TS=((“positive” NEAR/2 (affect* or emotion* or mood*))) #10. TS=((“self” NEAR/2 (concept* or perception* or “acceptance” or “confidence” or esteem* or “image” or “efficacy” or “reliance” or “worth” or “compassion”))) #11. TS=(((emotional* or event* or level* or “life” or perceiv*) NEAR/2 stress*)) #12. #1 OR #2 OR #3 OR #4 OR #5 OR #6 OR #7 OR #8 OR #9 OR #10 OR #11 #13. TOPIC: ((classroom* or highschool* or pupil* or school* or teacher* or “grade-1” or “grade one” or “first-grade” or “1st-grade” or “grade-2” or “grade two” or “second-grade” or “2nd-grade” or “grade 3” or “grade-three” or “third-grade” or “3rd-grade” or “grade-4” or “grade-four” or “fourth-grade” or “4th-grade” or “grade-5” or “grade five” or “5th grade” or “fifth grade” or “grade-6” or “grade-six” or “6th grade” or “sixth grade” or “grade-7” or “grade-seven” or “7th grade” or “seventh grade” or “grade-8” or “grade-eight” or “8th grade” or “eight grade” or “grade-9” or “grade-nine” or “9th grade” or “ninth grade” or “grade-10” or “grade-ten” or “10th grade” or “tenth grade” or “grade-11” or “grade-eleven” or “11th grade” or “eleventh grade” or “grade-12” or “grade-twelve” or “12th grade” or “twelfth grade”)) #14. TOPIC: ((adolescen* or “boys” or child* or “girls” or juvenil* or minor* or offspring* or “puberty” or school-age* or teen* or young* or youth* or underage* or “under age*”)) #15. #13 AND #14 #16. TS=(((classroom* or highschool* or pupil* or school* or teacher*) NEAR/2 (“based” or environment* or intervent* or implement* or setting*))) #17. TS=((school* NEAR/1 (“elementary” or “high” or “middle” or “primary” or “secondary”))) #18. #17 OR #16 OR #15 #19. TOPIC: (((cross-agenc* or crossagenc* or “cross-disciplinar*” or crossdisciplinar* or “cross-institutional*” or crossinstitutional* or “cross-organi?ational*” or crossorgani?ational* or “cross-professional*” or crossprofessional* or crosssectoral* or “cross-sectoral*” or “inter-agenc*” or interagenc* or “inter-disciplinar*” or interdisciplinar* or “inter-institutional*” or interinstitutional* or “inter-organi?ational*” or “interorgani?ational*” or “inter-professional*” or interprofessional* or “inter-sectoral*” or intersectoral* or “multi-agenc*” or multiagenc* or “multi-disciplinar*” or multidisciplinar* or “multi-institutional*” or multiinstitutional* or “multi-organi?ational*” or multiorgani?ational* or “multi-professional*” or multiprofessional* or “multi-sector*” or multisector* or “trans-agenc*” or transagenc* or “trans-disciplinar*” or transdisciplinar* or “trans-institutional*” or “transinstitutional*” or “trans-organi?ational*” or “transorgani?ational*” or “trans-professional*” or transprofessional* or “trans-sectoral*” or transsectoral*) NEAR/6 (“care” or collaborat* or communicat* or cooperat* or “health care” or intervention* or “mental health” or partnership* or program* or relation* or team* or strateg*))) #20. TS=((professional* NEAR/2 (collaborat* or coordinat* or cooperat* or partnership* or “teamwork”))) #21. TS=(((collaborat* or coordinat* or cooperat*) NEAR/5 (behavior* or behaviour* or “health” or intervention* or “mental health” or program* or “school* staff*” or “school* nurs*” or strateg*))) #22. #21 OR #20 OR #19 #23. TS=(((emergenc* or “health” or psychiat* or psycholog*) NEAR/2 (“care” or “nursing” or practice* or service*))) #24. TOPIC: ((“adolescent psychiatry” or “assertive community treatment” or “child guidance” or “child psychiatry” or “child welfare” or “community psychiatry” or “healthcare” or “mental health center*” or “mental health clinic*” or “mental health rehabilitation” or “primary care” or “psychiatric rehabilitation” or “psycholog* rehabilitation” or “psychosocial rehabilitation” or “social psychiatry” or “social service*” or “social work*” or support* or treat*)) #25. #24 OR #23 #26. TOPIC: ((barrier* or determinant* or effect* or evaluat* or facilitat* or factor* or implement* or intervent* or predict* or program*)) #27. #26 AND #25 AND #22 AND #18 AND #12978 #28. Refined by: [excluding] DOCUMENT TYPES: (PROCEEDINGS PAPER) AND LANGUAGES: (ENGLISH OR DANISH) 850	

### Appendix A.3. Psycinfo

Interface: Ovid Date of Search: 3 May 2019 Number of hits: 1238 Comment: In Ovid, two or more words are automatically searched as phrases; i.e., no quotation marks are needed	Field labels exp/= exploded controlled term /= non exploded controlled term .ti,ab,id. = title, abstract and author keywords adjx = within x words, regardless of order * = truncation of word for alternate endings
1. Mental Disorders/ 2. exp Mental Health/ 3. exp Psychopathology/ 4. Adjustment Disorders/ 5. exp Affective Disorders/ 6. “Depression (emotion)”/ 7. exp Anxiety Disorders/ 8. Anxiety/ 9. Fear/ 10. Panic/ 11. Performance Anxiety/ 12. exp Eating Disorders/ 13. Obsessive Compulsive Personality Disorder/ 14. exp Compulsions/ 15. exp Obsessions/ 16. Impulsiveness/ 17. Aggressive Behavior/ 18. exp Self-destructive Behavior/ 19. exp Somatoform Disorders/ 20. exp Sleep Disorders/ 21. Headache/ 22. Chronic Pain/ 23. Back Pain/ 24. exp Attention Deficit Disorder/ 25. Hyperkinesis/ 26. Social Issues/ 27. exp Behavior Disorders/ 28. Antisocial Personality Disorder/ 29. exp Drug Usage/ 30. Psychological Stress/ 31. “Quality of Life”/ 32. Satisfaction/ 33. Happiness/ 34. Pessimism/ 35. Self-concept/ 36. exp Body Image/ 37. Self-efficacy/ 38. ”Sense of Coherence”/ 39. exp Adjustment/ 40. “Resilience (psychological)”/ 41. ((mental or emotional) adj3 (disease* or disorder* or distress or health or illness* or ill health or illhealth or instabilit* or problem* or symptom*)).ti,ab,id. 42. ((psychiatric or psychologic*) adj3 (disorder* or distress or ill health or illhealth or illness* or problem* or symptom*)).ti,ab,id. 43. ((behavior or behaviour) adj2 (disorder* or problem* or symptom*)).ti,ab,id. 44. (affective adj2 (disorde* or distress or problem* or symptom* or syndrome*)).ti,ab,id. 45. (abdominal pain or abnormal psychology or adhd or adjustment disorder* or acrophobia or aggression or aggressive* or agoraphob* or anankastic or anorectic or anorexia* or antisocial or anxiety or anxious or attention defici* or attention problem* or avoidance or avoidant disorder* or back ache or back pain or binge eating or bulimi* or chronic pain or claustrophobia or compulsion or compulsiveness or compulsive or concentration problem* or delinquenc* or delinquent or depress* or despair or despondency or dysthymi* or eating problem* or externalising or externalizing or fear or frustrat* or headache or health complaint* or hopeless* or hyperactiv* or hyperkines* or impuls iv* or inattention or insomnia or internal distress or internalising or internalizing or intrusive thinking or intrusive thoughts or loneliness or lonely or mood or moods or musculoskeletal pain or musculoskeletal symptom* or neophobia or nervousness or norm breaking or obsess* or ophidiophobia or overanxious or pain disorder* or panic attack* or panic disorder* or perpetrator* or persistent pain or pessimism or pessimistic or phobia or phobic or psychopathology or psychosocial or psychosomatic* or recurrent pain or rule breaking or sadness or sad or school phobia or self-cut* or self-destructive* or self-harm* or self-injur* or suicide or sleep disturbance or sleep problem* or stomach ache or stomachache or social problem* or social withdrawal or unhapp* or worries or worry).ti,ab,id. 46. ((alcohol or appetite or body dysmorphic or body image or cyclothymic or child reactive or dysthymic or eating or factitious or feeding or impulse control or sleep or somatoform or substance) adj1 disorder*).ti,ab,id. 47. ((alcohol* or drug* or substance* or tobacco) adj3 (abuse or addiction or dependence or habituation or misuse or “use”)).ti,ab,id. 48. (well-being or wellbeing or wellness or optimis* or cheerful* or contentment or elated or elation or joy or enjoyment or good feeling* or good mood or happiness or happy or satisfaction or quality of life or HRQoL or QoL or sense of coherence or resilience or coping).ti,ab,id. 49. (positive adj3 (affect* or emotion* or mood*)).ti,ab,id. 50. (self adj3 (concept* or perception* or acceptance or confidence or esteem* or image or efficacy or reliance or worth or compassion)).ti,ab,id. 51. ((emotional* or event* or level* or life or perceiv*) adj3 stress*).ti,ab,id. 52. or/1–51 53. Schools/ 54. (classroom* or highschool* or pupil* or school* or teacher* or grade-1 or grade one or first-grade or 1st-grade or grade-2 or grade two or second-grade or 2nd-grade or grade 3 or grade-three or third-grade or 3rd-grade or grade-4 or grade-four or fourth-grade or 4th-grade or grade-5 or grade five or 5th grade or fifth grade or grade-6 or grade-six or 6th grade or sixth grade or grade-7 or grade-seven or 7th grade or seventh grade or grade-8 or grade-eight or 8th grade or eight grade or grade-9 or grade-nine or 9th grade or ninth grade or grade-10 or grade-ten or 10th grade or tenth grade or grade-11 or grade-eleven or 11th grade or eleventh grade or grade-12 or grade-twelve or 12th grade or twelfth grade).ti,ab,id. 55. Or/53–54 56. (adolescen* or boys or child* or girls or juvenil* or minor* or offspring* or puberty or school-age* or teen* or young* or youth* or underage* or under age*).ti,ab,id. 57. 55 and 56 58. Elementary Schools/ 59. High Schools/ 60. Junior High Schools/ 61. Middle Schools/ 62. School Based Intervention/ 63. School Nurses/ 64. ((classroom* or highschool* or pupil* or school* or teacher*) adj3 (based or environment* or intervent* or implement* or setting*)).ti,ab,id. 65. (school* adj2 (elementary or high or middle or primary or secondary)).ti,ab,id. 66. or/57–65 67. Work Teams/ 68. Interdisciplinary Treatment Approach/ 69. Collaboration/ 70. Cooperation/ 71. exp Group Performance/ 72. Collective Behavior/ 73. Integrated Services/ 74. ((cross-agenc* or crossagenc* or cross-disciplinar* or crossdisciplinar* or cross-institutional* or crossinstitutional* or cross-organi#ational* or crossorgani#ational* or cross-professional* or crossprofessional* or crosssectoral* or cross-sectoral* or inter-agenc* or interagenc* or inter-disciplinar* or interdisciplinar* or inter-institutional* or interinstitutional* or inter-organi#ational* or interorgani#ational* or inter-professional* or interprofessional* or inter-sectoral* or intersectoral* or multi-agenc* or multiagenc* or multi-disciplinar* or multidisciplinar* or multi-institutional* or multiinstitutional* or multi-organi#ational* or multiorgani#ational* or multi-professional* or multiprofessional* or multi-sector* or multisector* or trans-agenc* or transagenc* or trans-disciplinar* or transdisciplinar* or trans-institutional* or transinstitutional* or trans-organi#ational* or transorgani#ational* or trans-professional* or transprofessional* or trans-sectoral* or transsectoral*) adj6 (care or collaborat* or communicat* or cooperat* or health care or intervention* or mental health or partnership* or program* or relation* or team* or strateg*)).ti,ab,id. 75. (professional* adj3 (collaborat* or coordinat* or cooperat* or partnership* or teamwork)).ti,ab,id. 76. ((collaborat* or coordinat* or cooperat*) adj6 (behavior* or behaviour* or health or intervention* or mental health or program* or school* staff* or school* nurs* or strateg*)).ti,ab,id. 77. or/67–76 78. exp Child Welfare/ 79. Child Psychiatry/ 80. Adolescent Psychiatry/ 81. Health Care Services/ 82. exp Mental Health Services/ 83. exp Community Services/ 84. Psychiatric Nurses/ 85. Community Psychiatry/ 86. exp Social Casework/ 87. Primary Health Care/ 88. ((emergenc* or health or psychiat* or psycholog*) adj3 (care or nursing or practice* or service*)).ti,ab,id. 89. (adolescent psychiatry or assertive community treatment or child guidance or child psychiatry or child welfare or community psychiatry or healthcare or mental health center* or mental health clinic* or mental health rehabilitation or primary care or psychiatric rehabilitation or psycholog* rehabilitation or psychosocial rehabilitation or social psychiatry or social service* or social work* or support* or treat*).ti,ab,id. 90. or/78–89 91. exp Program Evaluation/ 92. Exp Treatment Effectiveness Evaluation/ 93. (barrier* or determinant* or effect* or evaluat* or facilitat* or factor* or implement* or intervent* or predict* or program*).ti,ab,id. 94. r/91–93 95. 52 and 66 and 77 and 90 and 94 1642 96. 95 not (exp animals/not humans.sh.) 1641 97. 96 and (danish or english or norwegian or swedish).lg. 1557 98. 97 not dissertations.dt. 1238	

### Appendix A.4. ERIC

Interface: ProQuest Date of Search: 3 May 2019 Number of hits: 1231	Field labels MAINSUBJECT.EXACT.EXPLODE = exploded subject heading MAINSUBJECT.EXACT non exploded subject heading TI,AB = title, abstract N/x = within x words, regardless of order * = truncation of word for alternate endings
(MAINSUBJECT.EXACT(“Mental Health” OR “Mental Disorders” OR “Psychopathology” OR “Adjustment (to Environment)” OR “Affective Behavior” OR “Depression (Psychology)” OR “Anxiety” OR “Fear” OR “Eating Disorders” OR “Aggression” OR “Sleep” OR “Pain” OR “Hyperactivity” OR “Social Problems” OR “Delinquency” OR “Antisocial Behavior” OR “Quality of Life” OR “Well Being” OR “Wellness” OR “Life Satisfaction” OR “Psychological Patterns” OR “Resilience (Psychology)”) OR MAINSUBJECT.EXACT. EXPLODE(“Anxiety Disorders” OR “Self Destructive Behavior” OR “Emotional Disturbances” OR “Attention Deficit Disorders” OR “Behavior Disorders” OR “Substance Abuse” OR “Self Concept”) OR TI,AB((mental OR emotional OR psychiatric OR psychologic*) N/3 (disease* OR disorder* OR distress OR health OR illness* OR “ill health” OR illhealth OR instabilit* OR problem* OR symptom*))) AND (TI,AB((classroom* OR highschool* OR pupil* OR school* OR teacher*) N/3 (based OR environment* OR intervent* OR implement* OR setting* OR elementary or high or middle or primary or secondary)) OR (MAINSUBJECT.EXACT.EXPLODE (“Students” OR “Schools”) OR MAINSUBJECT.EXACT(“Educational Environment” OR “Classroom Environment” OR “School Health Services” OR “School Nurses” OR “Ancillary School Services”) )) AND ((MAINSUBJECT.EXACT(“Interprofessional Relationship” OR “Interdisciplinary Approach”) OR MAINSUBJECT.EXACT.EXPLODE(“Cooperation”)) OR TI,AB(collaborat* OR coordinat* OR cooperat* OR partnership* OR teamwork) OR TI,AB((“cross-disciplinar*” OR interagency* OR interdisciplinar* OR intersectoral* OR interinstitutional* OR interprofessional* OR multidisciplinar*) N/6 (care OR communicat* OR “health care” OR intervention* OR “mental health” OR program* OR relation* OR team* OR strateg*))) AND (MAINSUBJECT.EXACT(“Child Welfare” OR “Health Services” OR “Community Health Services” OR “Clinics” OR “Psychoeducational Clinics” OR “Psychiatric Services” OR “Psychological Services” OR “Social Work” OR “Primary Health Care”) OR TI,AB((emergenc* OR health OR psychiat* OR psycholog*) N/3 (care OR nursing OR practice* OR service*)) OR TI,AB(healthcare OR “mental health center*” OR “mental health clinic*” OR “mental health rehabilitation” OR “primary care” OR “psycho* rehabilitation” OR “social service*” OR “social work*” OR support* OR treat*)) AND (MAINSUBJECT.EXACT(“Program Evaluation” OR “Program Effectiveness” OR “Program Development” OR “Program Implementation” OR “Mental Health Programs”) OR TI,AB(barrier* OR determinant* OR effect* OR evaluat* OR facilitat* OR factor* OR implement* OR intervent* OR predict* OR program*)) **Applied filters:** Scholarly Journals OR Reports OR Dissertations and Theses	

## Appendix B. Eligibility Criteria

**Study Focus/Content**	**Inclusion Criteria**	**Exclusion Criteria**
**Implementationof the** **intervention**	Studies that describe: - Determinants, challenges, problems, barriers, supportive factors that are - linked to the characteristics of the intervention - linked to the outer context - linked to the inner context - linked to the characteristics of the individuals - linked to the implementation process	Studies that describe: - Prevalence of illness - Needs of interventions - Factors/determinants that are predicted and not demonstrated
**The Interventions**	- Mapping i.e., identify symptoms and causes - Assessment of what type of intervention that is needed - Health promoting intervention i.e., highlights and strengthens what works for the individual - Interventions to ensure that health promoting interventions reach children at risk - Preventive interventions - Referrals of acute or extensive needs - Support/treatment i.e., all interventions that intend to increase wellbeing - Consult support to other organisations i.e., to educate about mapping, support, and referrals - Offer the right help at the right time i.e., availability and early discovery - Child perspective i.e., the child’s best at interest and to involve the child - Support for the transition into adulthood	- Studies about ongoing work and not specific interventions
**Collaborative partners**	- Social worker/representative from the social services - Employed within the health care services - Employed at a university with a clinical role	- Only a school or collaboration with other schools - A university with researchers without clinical roles - Non-profit organisations - Voluntary work - Student interns from vocational education - Studies with more collaborative partners than schools, social services and health care services
**Arena of the intervention**	- In school (preschool to upper secondary school) not necessary with school personnel - With a school representative with a specific role to collaborate externally	- Outside school and without a school representative - Education outside school, for example juvenile facilities/jail - Interventions conducted solely outside of school
**Target group**	- Children and youth with signs of or at risk of mental ill-health - People close to the child, i.e., people who work with children or caregivers	- Studies that include other groups outside our target group such as college students or infants
**Problem**	- Mental ill-health - Indicators of mental ill-health	- Studies that describe interventions targeted more problems than mental ill-health, symptoms of mental ill-health or indicators of mental ill-health. For example, interventions that promote availability of care targeting general health and not only mental ill-health
**Language**	- English - Scandinavian languages	
**Type of publication**	- Scientific journals - Reports - Dissertations	- Conference abstracts - Study protocols - Reviews - Debate articles - Books - ’’Case’’studies about single individuals
